# The Bristol Infirmary in My Student Days, 1822—1828

**Published:** 1890-09

**Authors:** Henry Alford

**Affiliations:** Consulting Surgeon to the Taunton and Somerset Hospital


### THE BRISTOL INFIRMARY IN MY STUDENT DAYS, 1822—1828.

BY

HENRY ALFORD, F.R.C.S.

*Consulting Surgeon to the Taunton and Somerset Hospital.*

---

I WAS elected a house-pupil, or apprentice, to the Infirmary, and began my residence there on March 19th, 1822, being then 15 years and 8 months of age.

The house-pupils were then elected by the subscribers or trustees; and if there was more than one candidate, there was a canvass, and election by voting.

We were apprenticed for five years to the Committee or Governing Body of the Infirmary, and to the resident medical officer for the time being. He was then called the house-apothecary. The fees paid by my father were 150 guineas to the treasurer of the Infirmary as a premium: 30 guineas a year for lodging, boarding, and washing, and 40 guineas as a fee or premium to the house apothecary. There were three of these house-pupils; and as the situation was in high repute, vacancies were always filled quickly, and sometimes contested. There were fifteen surgeons' pupils, each of the five surgeons being limited to three. These were selected or chosen by the surgeons themselves; but each pupil had to sign a declaration of conformity to the rules and regulations before he was allowed to attend the practice of the Infirmary, which was a large, well-appointed provincial hospital, making up 200 beds for in-patients, and claiming to be the oldest provincial hospital in England. It was also the only general hospital in Bristol. The pupils had abundant opportunities of getting a good practical knowledge of medicine, surgery, and anatomy.

The in-patients were about equally divided into medical and surgical cases; the former being in the wards on the first floor, the latter on the second. Each floor was divided in the middle by strong iron gates, which were always left locked at night; female patients being on the one side, and males on the other. There was an average of about sixteen accidents or casualties every week. These were always admitted at once, without any recommendation. Many of them were severe and complicated, and some required immediate consultation or operation. When this was the case, the surgeon for the week, or all the surgical staff were summoned at any time, day or night. One pupil of the surgeon for the week was in charge of casualties, and was always summoned for every case, however slight, although he did not sleep in the house; and it was left to his judgment to summon the surgeon for the week. The resident medical officer and his pupils had nothing officially to do with the treatment of the surgical patients. The surgeons were very jealous of any interference on their part. Notwithstanding this feeling, however, it was very common for the pupil in charge to ask one of the house-pupils to act for him at night, and sometimes by day, after the usual visit and daily work of the Infirmary were over. We house-pupils were always ready to undertake this work, and I got a great amount of practice in the treatment of wounds and the slighter injuries in this way during my apprenticeship. The care of the medical wards, involving the treatment of medical cases, was the special charge of the resident medical officer, under the physicians. He went regularly round the medical wards, seeing every patient, every morning after breakfast, his two senior pupils going with him, and also some of the surgeons' pupils, who paid a special fee for that privilege. The resident medical officer, or one of his pupils, always attended the physicians in the out-patient room and when they visited the wards, writing their prescriptions, either in books for the in-patients, or on pieces of paper for the out-patients. The junior house-pupil was chiefly engaged in the dispensary or in keeping the books, and giving the matron an account of the changes of diet, &c., which he took down in all the wards every morning. He had, however, the privilege of seeing all operations and *post mortem* examinations.

There were frequent operations at the Infirmary of almost every kind. As many acute and fatal cases were admitted, and there was an autopsy in nearly every case of death, there were abundant opportunities of getting a practical knowledge of all the viscera, and also admitting of superficial dissections of many parts without any apparent injury or disfigurement of the body. It will be evident from this sketch that a diligent pupil, who kept his eyes open and his hands and fingers well employed, had a grand opportunity of learning all the practical details of his profession.

As in those days the heroic method of treatment was in the ascendant, and many drugs were used, the dispensing department of the Infirmary was a very important one. For this work there were a dispenser (called Cross), a laboratory man, and a shop-boy, besides the house pupils, who were all supposed to assist in the dispensary; but only the junior had his chief daily work in the dispensary. Nearly all the preparations for dispensing, and many of the chemicals, were made in the laboratory.

There was no Medical School in Bristol in my time. A course of lectures on anatomy was given every winter by Dr. Wallis, in a house in Lower College Green, assisted at a later date by Dr. Riley. These lectures were attended by several of the Infirmary pupils; I attended some of them. I remember one winter we supplied a sufficient number of subjects for dissection from the Infirmary dead-house, by removing the bodies from the coffins and putting in some substitute. There were also occasional lectures on chemistry and botany given by Mr. Herapath. At that time the College of Surgeons did not recognise the certificates of these lectures, or the medical or surgical practice of the Infirmary. But the Apothecaries' Hall admitted both.

There were two nurses to each of the wards, a day and night nurse; the former was the superior, and in charge of the ward, and sat up until 11 or 12 o'clock each night, after which the night nurse took charge of the ward for the rest of the night. The night nurse did most of the cleaning and scrubbing and drudgery of the ward. Most of these were quite young and inexperienced women. Many of the day nurses were well qualified and efficient, judged according to the requirements of a nurse at that time. All the nurses were of the class of domestic servants, and there was no head nurse. All were under the superintendence and authority of the matron, who had also, I believe, the full power of appointment and dismissal.

The house-apothecary and three house-pupils had their meals with the matron (Mrs. Davey), in her sitting-room, which was also her store-room for linen, &c., and from which her bedroom opened. The pupils had no other sitting-room except their bedrooms, and these were not private. For some time after I went to the Infirmary, I slept in a double-bedded room with a fellow pupil (J. Butter). The medical officer had a separate sitting-room and bedroom adjoining. All these rooms were on the ground-floor, on a level with the entrance-door.

The medical and surgical staff of the Infirmary consisted of four physicians, five surgeons, and the resident medical officer or house-apothecary. I will record some recollections of these in succession, and of their method of treatment and practice.

But, as in duty bound, I will give first a slight sketch of my immediate master, Mr. William Swayne. He was not a very young man, over 30, I should think, when I first knew him. He was a very quiet, silent man, very clever, and very well up in his profession. He had little intercourse with his pupils, except in the way of their work; and we saw very little of him, except at meals and in going round the wards with him in the morning, which privilege I did not enjoy during my first year. All the medical and surgical staff had a high opinion of Mr. Swayne's abilities, and often took him to see their private patients with them. He was a very sedate, self-reliant, gentlemanly man, and, I should think, of superior general knowledge and culture. But he had some sense of dry humour, which showed itself occasionally at our meals. He was very regular and moderate in his eating: for his breakfast he always had coffee, one boiled egg, and two slices of bread and butter, which one of his pupils cut for him. He scarcely ever spoke at meals, except to ask or answer a question. But he smiled occasionally at any amusing remark or matter that struck his fancy.

Mr. W. Swayne had an elder brother in practice in

Bristol, who had a high reputation in midwifery practice. Two or three years after the commencement of my apprenticeship, poor Mr. Swayne had symptoms of incipient phthisis. He was sent a voyage to the West Indies, a common practice in such cases at that time, but returned without any durable benefit, and died at the end of a year. Mr. W. F. Morgan held the situation for him until his death, and was appointed as his successor. He was my first cousin, and some years after my brother-in-law, and was subsequently surgeon and consulting surgeon to the Royal Infirmary. He must have been personally known to many of the present members of the profession in Bristol and Clifton, as he died in December, 1872.

I will now try to give a slight sketch of each of the physicians and surgeons in order. This may be interesting to the present generation of the profession. Indeed, so great is the change in the theory and practice, both of medicine and surgery, since the date of which I am writing, that my record has almost an archæological interest.

The physicians and surgeons had each and all a marked individuality of character and practice. The observation of their different modes of treatment, and their results, especially of the medical cases, by the physicians, was not the least useful part of the medical education of the pupils. That was my experience.

Dr. Andrew Carrick was the senior physician. He was a tall, sedate Scotchman, grave and gentlemanly in appearance and bearing; kind and gentle and pains-taking with his patients at the Infirmary, with the out-patients as well as with those in the house. He would take from one to two hours with his out-patients, on the two days of each week on which he attended, to see and prescribe for them, which he very rarely missed, sometimes trying the patience of the resident medical officer or his pupil, who sat by his side and wrote his prescriptions on slips of paper, and also of those who waited to dispense them. His treatment was essentially of the depleting and lowering character. He was specially a great bleeder. The little scraps of paper with V.S. ad. ℥xij., ℥xvj., ℥xx., &c., often amounted to ten, fifteen, or twenty on his out-patient days, keeping the pupil for the week pretty fully occupied. He also prescribed purgatives, mercurials, and nauseating medicines very freely. Every acute case admitted into the house under his care was pretty sure to be bled freely, and repeatedly, if the blood was buffed or cupped, purged by calomel and colocynth and black draught, followed by saline antimonial mixtures every four hours, and a low slop diet. The same treatment, with little variation, was adopted in cases of fever (which were then freely admitted into the general medical wards and mixed with the other cases); and in cases of acute rheumatism, as well as in all cases of acute inflammations. Dr. Carrick went round the wards to see his in-patients with a long, serious, stately stride, spoke always gently and kindly to his patients, and occasionally interposed some little word of religious comfort or advice. I think he was generally a favourite with the pupils, except that some of them complained of the work he gave them in the bleeding line, and the senior house-pupil was sometimes impatient of his long attendance on him with his in- or out-patients. Dr. Carrick had a large private and consulting practice. He lived in a large house at Clifton, near the Down I think. He drove a somewhat old-fashioned chariot, with a pair of steady old horses driven by an old coachman. He always came to the Infirmary in his carriage, and many a weary hour his old horses and coachman must have had to wait for him in the street. Dr. Carrick was a well-informed man in general literature, as well as in his profession. He was kind and courteous to all, and was, I think, generally liked. He was in the habit of inviting all, or a large part, of the medical men of Bristol and Clifton to a dinner-party at least once a year. He kept up the observance of St. Andrew's Day by a dinner-party. From his occasional serious words to his Infirmary patients, he was, I believe, a good Christian man, as well as a kind and courteous gentleman. He attended Mrs. Hannah More in the latter part of her life, and was, I think, a personal and valued friend of hers.

The next physician in order of seniority was Dr. J. E. Stock. He was in appearance and manner and mode of practice a complete contrast to Dr. Carrick. He was of middle age, rather short and slight, somewhat loosely dressed, with a slightly shambling walk, and a rather indecisive manner and address. He was, I think, rather short-sighted, and had a habit of half closing his eyes, or pushing up the lower lid of one of them, when he specially looked at any person or object. He was a man of literary taste and acquirements, and he associated with persons of that character and reputation. Amongst others, he was very intimate with Mrs. Skimmelpennick, who then lived in a house a few hundred yards from the Infirmary. He had also, I think, some intimacy and connection with a congregation of Moravians, who had a chapel near the Infirmary.

His medical opinions and practice were peculiar for that day. He appeared to act on the belief that nearly all deviations from health arose from want of vital power or deficiency of food. He carried this out in his own personal habits, generally carrying in his pockets some of Abernethy's biscuits, or other light food, which he often munched as he was going round the wards, or in the out-patient room. Of course he carried out this system in the treatment of his patients. His prescriptions were nearly all composed of tonics or stimulants, to which was added a full nutritious diet. He used, very sparingly, the almost universal depleting treatment of the day: bleeding, purgatives, nauseating mixtures, &c., even in the cases of acute inflammation.

I have, however, a very vivid recollection of one unfortunate prescription of his, the effect of which left a strong prejudice on my mind against the medicine he then prescribed, when it was re-introduced many years after, and soon became in general use. Dr. Stock had two male patients in one ward suffering from chronic rheumatism. In the treatment of these cases the ordinary remedies had failed to give relief. One day the following prescription, in Dr. Stock's book, for both these patients, was brought to the dispensary: ℞ Ol. Jecoris Aselli, coch. mag. iij. ter di. None of us pupils could make it out: we had never heard of such a drug; but the dispenser (Cross), after some searching, brought out from the store-room an old-fashioned black quart bottle, covered with dust and cobwebs, three-parts full of a dark-coloured oil, with a strong, fishy, and somewhat rancid smell. The faded label on the bottle corresponded with the medicine prescribed. No one knew how long the bottle had been in store. There was no record of its having been used within living memory. However, two eight-ounce bottles were filled with this oil, labelled according to the prescription, and sent to the ward, and some of the contents taken by the patients as directed. I do not remember how many doses were taken, or whether they were retained on the stomach; but on the following day both the patients were attacked with acute gastritis. One of them died a day or two after; the other recovered. The autopsy of the fatal case showed an intensely inflamed state of the whole mucous membrane of the stomach, with dark-red, almost black, patches of the size of a sixpence scattered over the surface of it, having almost the appearance of extravasation of blood. I write chiefly from memory, and I do not remember the after-history of the patient who recovered from the gastritis, nor whether his rheumatism was relieved or removed. Dr. Stock lived at Clifton; I think his private and consulting practice was not large. He attended the Infirmary pretty regularly, and always drove there in a carriage and pair. He was sociable and friendly with the pupils, and rather liked by them, but often laughed at and quizzed for his peculiarities.

The next physician in seniority, Dr. Henry Hawes Fox, was quite different in appearance, manners, and practice from the two former. He was pre-eminently a courtier, with soft, slow, and gentle speech, and kind and insinuating look and manner. A rather stout, handsome, middle-aged man, always well dressed. His walk was quiet and deliberate, and apparently slow. But, with all that, he lost no time in getting through his work and over the ground he had to go. The treatment of his patients at the Infirmary was almost entirely expectant; he ordered scarcely any medicines but mild sedatives and placebos. Dr. Fox's chief aim in seeing his in- and out-patients at the Infirmary was, apparently, to get through the work as quickly as he could, and yet in the most courteous and deliberate manner. He would see and prescribe for twenty to forty or fifty out-patients in twenty or twenty-five minutes, and would go round the wards in the same time and manner.

Seated by the table in the out-patient room, with the resident medical officer or his senior pupil by his side to write his prescriptions, this would be the general mode of procedure. As they came into the room, one at a time, and stood (rarely sat) by his side, Dr. Fox would look kindly and say, "How—do—you—do, my friend?" Some answer would come from the patient; but the doctor would immediately turn to his attendant scribe, and say, "Continue," or "Repeat, sir"; and then to the boy at the door, "Next, if you please." So rapid was he in getting through his list of patients in this manner, that it was difficult to keep up with him in writing, although there were rarely any fresh prescriptions, except for new patients. Dr. Fox's mode with his in-patients was much the same. He walked through the ward, and from bed to bed, with slow, deliberate, gentle steps, scarcely ever stopping at a bed more than a few seconds, but always addressing each patient with a slow and courteous enquiry as to his or her health, and often saying, "Good-day to you," or "Good-morning," as he passed on to the next bed. Dr. Fox was always very courteous to the resident medical officer and to the pupils. Indeed, from his manner and conduct at the Infirmary, one would infer that he could never be angry or say an unkind word; that he would never be in a hurry, and yet would get through his work in less time than any one else could. Dr. Fox had a large and fashionable practice, both private and consulting. He drove a handsome carriage, well appointed in every respect; he was very particular about his horses, which were usually grey. There was a story, however, that he or his coachman had once bought a new high-priced horse which, when brought to Dr. Fox's, appeared familiar with the stable, and, after a careful examination, was found to be the same old horse which had been sold a short time before as being worn-out, but which had been so doctored and transformed by the dealer as to deceive the knowing ones. I will not answer for the truth of this story; I know it only from hearsay.

Dr. J. C. Prichard, although the junior physician at the Infirmary in my time, was far in advance of the others in culture, in general and professional knowledge, and in literary reputation, which was not limited to Bristol or to England, but was European. He was a small, spare man, with a short, quick, decided step; sharp and somewhat curt in his speech, but kind and very attentive to his hospital patients. He generally wore a large, loose overcoat, with roomy side-pockets, large enough to hold a quarto or small folio case-book; and he generally carried other books with him on the seat of his carriage. He took notes of the cases of his patients in the Infirmary, in short, terse Latin sentences, in his case-book, which he always carried himself, and brought to the Infirmary and took away with him. He was a great linguist, and would talk to his patients, when necessary, in their own language, French, German, and especially Welsh, from which country we had many in the Infirmary, some of whom could hardly understand English. (I have heard that Dr. Prichard considered the Welsh the most musical and melodious of languages.) It was said that he had talked Hebrew with a Jew. I certainly did not hear this. The other languages mentioned I have myself heard.

Dr. Prichard's treatment of the cases under his care was, even then, considered heroic. He did not bleed so much as Dr. Carrick; but counter-irritation in every form he pushed to an extreme degree. Blisters, often very large ones, setons, issues made by caustics or the knife, and the suppuration kept up by peas or medicated beads. One issue was called by his name. An incision through the scalp from the vertex to the forehead, three or four inches in length, kept open, first by dry lint, and after suppuration was established, by peas, in cases of cerebral disease or mischief. I have certainly seen undoubted benefit from this mode of treatment in some severe cases.

Dr. Prichard's heroic treatment extended to the use of drugs. Mercury, digitalis (generally given in the form of infusion and in large doses), colchicum, and other potent drugs were often pushed to the extreme limit of safety in the treatment of acute diseases. I may give an account of his treatment of myself, as a fair example of his usual mode of practice. Shortly before the end of my apprenticeship, in my twenty-first year, I had an attack of continued fever of some form, not very severe, principally affecting the head, but without delirium. Dr. Prichard attended me with Mr. Morgan, the then resident surgeon. He directed me to be bled to twenty ounces one forenoon; the same evening he ordered twenty leeches to be applied to my temples; and the following morning ten grains of calomel to be taken in one dose. This last acted as an emetic, and I brought it up. Mercurials had always a specially depressing effect upon me, and I was anything but a strong or robust man at the time. However, I recovered well, and without any continued drawbacks, except some prolonged weakness.

Dr. Prichard was in great general esteem as a man of wide knowledge and high literary accomplishments. He had a large consulting practice; not, I believe, an extensive private one. He drove a carriage and pair, like all the other physicians. He appeared to be always reading in his carriage, and had generally a heap of books with him. His wife was a Miss Estlin, one of a highly cultivated and much esteemed Bristol family, and connected with the Stuckeys and Bagehots of Langport.

The results of the different modes of practice in the treatment of diseases of these four physicians was well worth the careful observation of the pupils. It certainly was a profitable part of my medical education and experience, and has been a useful guide to me in my medical practice in after life.

I do not remember that the results differed much in acute diseases. Cases of fever, mostly gastric or typhoid, although they were not so called then, were freely admitted into the medical wards without any separation from the other patients. Many of them died, but I do not think the proportion of the fatal cases was greater under the treatment of Drs. Carrick and Prichard than under Drs. Stock and Fox. The same may be said of acute inflammations, pleurisy, pneumonia, peritonitis, acute rheumatism, meningitis, &c. Many of these were admitted into the Infirmary, and a certain proportion of them were fatal. Nearly all of them were bled in the early stage by the orders of their physician, or the resident medical officer. My impression is that their recovery, or death, or serious secondary consequences, depended more upon the previous health and strength of constitution of the patient than on the mode of treatment.

But in chronic cases, and especially in such as were necessarily fatal, the different effects of the treatment were so marked that the least observant pupil could hardly avoid noticing them. This was specially well marked in cases of phthisis. Many of these were admitted into the Infirmary, and a large proportion remained there till the fatal termination. Of these Dr. Fox's patients especially, who had no medical treatment but mild opiates and placebos, with warm comfortable quarters and a suitable diet, lingered on from week to week and month to month, as if they set death at defiance. But those who were subjected to active treatment of any kind, whether by drugs or counter-irritants, or any experimental management, soon gave us an opportunity of studying the morbid effects of the disease in the dead-house. The same may be said in a less degree of other chronic diseases, as cancer, paralysis, and others not often or necessarily fatal; chronic rheumatism, chronic visceral diseases, &c. The more they were left to the effects of comfort and repose and a suitable diet, and the less they were drugged and victimised by caustic or knife, the longer they lived, or the sooner they recovered or were relieved.

I had not much to do with the surgeons during my apprenticeship, as the house-pupils were not allowed to see much of their practice, except in the case of operations and severe casualties, and when acting as *locum tenens* for the pupil in charge, which was a frequent privilege—but this was *sub rosâ*. We could only judge of their general treatment of their cases by their prescriptions and the gossip of their pupils.

All the five surgeons were, however, sufficiently distinct in character and appearance, and in their mode of operating.

The senior surgeon was Mr. Richard Smith. He had been surgeon to the Infirmary for many years before I went there, and continued as acting-surgeon until his sudden death, many years after I left. I was a pupil and dresser under him for a year after the end of my apprenticeship, and before finishing my education in London. He was a well-known personage in Bristol outside his profession. He was a big, burly man, with a round, florid face, large whiskers, a hearty, jovial manner, and rather loud, brusque voice. He was a boon companion in society, and a welcome guest at public and private dinners. He could tell a good story or anecdote, and, I believe, sing a comic song. His tales were often professional; and neither those nor some of his jokes or songs were quite suitable for the ears of ladies or clergymen, or non-professional precise men. But in those days a good deal of coarseness in language was not only tolerated, but looked for and enjoyed at public gatherings. I believe "Dick Smith," as he was generally called, was a very popular man in Bristol, both within and without the profession. He was a good surgeon for those days; rather careless in the treatment of his patients, whether in the wards or in the operating theatre. He was a bold, steady operator, but rather rough and reckless; and not very mindful of the feelings or state of his patient. He was very popular with the pupils and his colleagues, and, I think, rather a favourite with the Infirmary patients.

He had a good general private practice, and also an operating and consulting practice. He was in the habit of seeing patients at his own house of a morning, mostly young men, and dispensing for them in a very rough way from his own surgery. One of his pupils attended there for an hour or two every morning, to show the patients who came into Mr. Smith's consulting-room (they generally came by a back-door opening into Park Row—Mr. Richard Smith lived in Park Street), and to dispense the medicines prescribed, usually verbally, and mostly limited to powders, pills, or ointment, put up in a very rough and ready style. I attended for this purpose during some part of my pupilage with him.

Mr. Richard Smith had formed a museum of specimens of morbid structures and formations, and some of general anatomy. This museum was specially rich in specimens of calculi, most of which had been removed by lithotomy at the Infirmary. These were all put up most carefully by himself, making sections of them, and fixing them in shallow trays of white cardboard, with circles of ink drawn round each with a pair of compasses, and a short description and history added. I think there must have been nearly a hundred of these calculi. He employed his spare time whilst waiting for his morning patients in putting them up and arranging them. There was one rather gaunt and weird object in this museum. It was a large case with a glass door in front. In this case was hung the skeleton of a young man, who had been hanged for murder, and his body given to the surgeons of the Infirmary for dissection. In a fit of jealousy he had flung a stone at a young woman whom he was courting. The stone struck her on the back of the head, and, I suppose, fractured her skull. She was taken to the Infirmary under Mr. Smith's care, and, I believe, trephined, and in a few days died. The skeleton was well preserved and articulated, and hung up in this case; and by its side were hung the cords by which he had been hung and his hands tied, and also a book containing all the briefs and other manuscripts connected with his trial, and a full account of the crime. This book had been bound with the tanned skin of the murderer. On the outside of the cover was a label with a printed inscription, "Cutis vera Johannis" (I forget his other name). All this occurred before I went to the Infirmary. A large room on the ground-floor of the Infirmary was given up to this museum whilst I was there, and Mr. R. Smith spent many hours of the forenoon in this room. The museum was generally open to the faculty and pupils of the Infirmary, and, I think, to the profession generally. And visitors, female as well as male, were often admitted to see it.

Mr. Richard Smith drove a high gig with a large old horse, and an old coachman as big and burly as his master. In wet or cold weather the coachman was well cased in a bulky box great-coat, with voluminous capes.

Mr. Richard Smith was a married man, but he had no children. His wife was well known and highly esteemed in Bristol as a very benevolent lady, and an active, earnest member of the Evangelical Church party, with whom she was reported to have kept up a good deal of visiting at her own house. But it was understood that her husband was never, or very rarely, present at these parties. I believe they lived very amicably in the same house, but had each their different friends and pursuits. I have an idea that Mrs. R. Smith had a private carriage for herself.

Mr. Richard Smith was really a very kind and considerate man, especially to his pupils. He would occasionally keep them in roars of laughter with some of his professional stories and anecdotes, which he told and embellished in a very graphic and humorous manner.

Mr. Hetling was the surgeon next in seniority. He was, I think, older than Mr. Richard Smith, and was very different from him in person, appearance, and general and professional character. He was a slight, thin man, not very free or communicative with his pupils; regular in his visits to the Infirmary, and he took a good deal of pains with his patients there, as to their general treatment, prescribing more medicine for them than some of the other surgeons.

He was a very slow and rather clumsy operator. In cases of strangulated hernia he would slowly divide every possible layer or subdivision of cellular tissue or fascia on a director, sometimes to the number of seven or eight or more, before exposing the sac; and in opening this he used every possible precaution. But we observed that his cases of this class more frequently recovered, and more speedily, than those of some of the other surgeons, who were more bold and quick operators. Mr. Hetling always appeared clumsy and awkward in the use of the knife to the spectators; but there was no apparent nervousness or want of self-confidence.

Mr. Hetling lived in a house in the Royal York Crescent, Clifton. He had, I think, a good private practice. He drove a close carriage and pair in his professional visits; but he sometimes walked to the Infirmary. He was a married man, with a large family of sons and daughters, and I think they visited a good deal. I was once at a dance at their house. One of his sons, who was fully qualified as a surgeon, died during my pupilage. His youngest son (George) was a pupil to his father at the Infirmary in my time. He was two or three years younger than myself. Some years later he left the medical profession and became a High Church clergyman. He married a Taunton lady, and I met him here occasionally in later years. I met him in the streets of

Taunton two or three years ago, and had a chat with him about old Infirmary legends.

Mr. Lowe comes next in seniority. His father had been surgeon to the Infirmary before him. Mr. Lowe had a very strong and well-marked individuality of character. He was rather a tall and spare but muscular man, with a clean-shaved face, good looking, but with a determined and somewhat cynical expression. He was always well dressed, but in what would be considered now, or even then, an old-fashioned style. He often wore breeches and top-boots, the latter beautifully made and well polished, with very light-coloured and well-made tops. In this dress he walked to see his patients and visited the Infirmary. He kept no horse or carriage, and, I believe, never rode in Bristol or Clifton: he always walked to the Infirmary. He had very well-formed and tapering hands and feet, and was supposed to be rather proud of them, hence his style of dress. He was a great friend and ally of Mr. Richard Smith, although so different in appearance and character. He was very cynical and severe in his remarks and opinion on people in general, and often came to the Infirmary charged with the last piece of gossip or scandal, which he retailed to his friend Smith in his peculiar dry, sneering tone. At the time the *Lancet* was first started, it had, nearly every week, some bit of scandal or criticism against one or more of the leading London hospital surgeons. Lowe would come to the Infirmary primed with this, and read it aloud to Richard Smith or any of his other colleagues with great gusto.

Mr. Lowe was a *very good operator.* It was quite a treat to watch the manner in which he used the knife—with great dexterity and neatness, and with perfect precision and anatomical skill, and yet with great boldness. I have seen him, in operating for strangulated hernia, put the skin on the stretch with the thumb and forefinger of his left hand, and then, with the scalpel in his right, divide it and the layer of cellular tissue and fascia rapidly, without using forceps or director, then opening the sac by pinching up a little fold of it with the forceps. He was very fond of operating, and laid himself out for it. He would not, if he could avoid it, admit any surgical chronic cases under his care, which could be cured or removed by an operation, without getting the consent of the patient previously to this mode of treatment. This was specially the case with old or extensive ulcers of the leg.

Mr. Lowe was a very determined, as well as bold, operator. I will relate a case in illustration of this. A young man was admitted under him, with reported calculus in the bladder, for lithotomy. He was examined with a sound. A consultation was held on his case, and, although there was some obscurity in the results of the sounding, all the surgeons agreed that there was a calculus, and that it was a proper case for lithotomy. Mr. Lowe operated, and opened the bladder in his usual prompt and skilful manner; but when the forceps were introduced, no stone could be found. Mr. Lowe was not to be baffled; he was confident that his diagnosis was right, and he proceeded to explore, and wash out the bladder, by every means he could devise, for a long time, but in vain. The patient was at last released and taken back to his bed, much exhausted. He died the next morning. His father was very angry and outrageous, and refused to allow an examination of the body, which he removed from the Infirmary the same day. It was reported that some surgeon, not connected with the

Infirmary, had made an examination of the body, and found a thickening and cartilaginous change of structure of some part of the coats of the bladder, which had led to the error in diagnosis.

Mr. Lowe did little in the way of the medical treatment of his patients. He was a strict disciplinarian with his pupils, required them to dress all wounds, sores, burns, &c., themselves, and to leave nothing to the nurses. He punished them pretty severely with his rough, sarcastic tongue, if he found them shirking their work. He was also very ready to put down anything like conceit or presumption in any of the pupils, whether his own or others, by some short, sharp, cutting remark. But he was admired, and, in a certain sense, liked by the pupils generally for his skill, and almost dramatic perfection, in the use of the operating knife.

Mr. Lowe had a small, but select and good, private practice, and, I think, some consulting operations. He was a married man with a family; but was reported to be somewhat cynical and reserved in his domestic relations, and to take his breakfast always alone in his consulting-room. He was said to be specially fond of strong green tea, which he kept for his special use. He lived to an advanced age, and, I think, continued acting as senior surgeon to the Infirmary long after the death of his friend, Richard Smith. Mr. Lowe presided at a dinner of old Infirmary men at the Montague Tavern, Kingsdown, some years after I was in practice at Taunton. I had engaged to attend it, but was prevented doing so at the last by some professional engagement.

Mr. Daniel was the next surgeon. He also had his peculiarities and individuality of character. He was a stout, good-looking, well-dressed man ; rather loud and positive in expressing his opinions and laying down the law. He was active in his medical as well as surgical treatment, prescribed freely active and powerful medicines for his Infirmary patients, and he went in strongly for the heroic treatment of that time. He was a rough and clumsy operator, and had not a clever use of his hands in surgical manipulations; but he had plenty of boldness and confidence in the use of the knife. He was inclined to be rough with his pupils, and almost bullied the younger ones sometimes if they neglected the patients; but he was considerate and friendly in his general manner and bearing to them. He was kind in taking notice of me, when I went first to the Infirmary, and gave me a hint or two, in his rough way, which were of much use to me in after life. I was not always correct in my spelling at that time. Some of my writing must have come under his notice in which I had made mistakes of this kind. He took an early opportunity of giving me some advice on the subject, somewhat in this way, you know: "Alford, a man may write illegibly without losing caste; but no gentleman spells badly." I date to that kind reproof my efforts to be more careful and correct in this matter.

Mr. Daniel had a very good general private practice—I think, at that time, the largest and most lucrative in Bristol. He drove a handsome carriage with a fine pair of horses, altogether a stylish turn-out. He had one daughter, I think his sole family. She afterwards married Dr. Riley.

Mr. Nathaniel Smith (commonly called "Nat Smith") was the junior surgeon. He was a short, bright, natty little man; very pleasant in manners and conversation; not very punctual or regular in his visits to the Infirmary, leaving a good deal to his pupils and to the resident medical officer. He was a *very good* operator, probably as good as Mr. Lowe, but somewhat different in his style of operating. He was specially neat and clean and precise in the use of the knife, without the dash and boldness of Mr. Lowe; but, perhaps, the safer operator. He never lost his perfect steadiness and presence of mind. I remember seeing him operate in a case of strangulated hernia, in which there was some difficulty and complication. There was some delay, and a doubt whether the intestine had not been wounded. Although he became pale, and beads of perspiration stood on his brow—I suppose from anxiety, and the length of the operation—yet his hand was perfectly steady and firm, and his manner and voice betrayed no anxiety or want of self-confidence.

Mr. Nathaniel Smith drove a smart carriage and pair, and generally came to the Infirmary in it. He had a good private practice, and frequent consultations and private operations, I believe. He had a wife and large family. He was the only one of the surgeons of the Infirmary at that time who took pupils in his own house. They were supposed to be under very little restraint or control, and some of them were not noted for steadiness or hard work.

Mr. Nathaniel Smith was the oldest survivor of the surgeons of my time. He lived at Weston-super-Mare after he had left the Infirmary, and given up private practice, except consultations and operations. He has seen patients of mine at Weston in consultation with me. I have heard of his operating at Weston when he must have been eighty years of age, or nearly as much, without failure of sight or unsteadiness of hand. He died at Weston some years ago at an advanced age, much beyond eighty.

Before concluding these reminiscences, I will record two or three incidents peculiar to that time which stand out clear in my memory. From the general adoption of heroic practice in the treatment of disease there was a great deal of bleeding, not only from the arm, but also by cupping, opening the temporal artery or jugular vein, and by leeches. All these minor operations were performed by the pupils. The number of bleedings on Dr. Carrick's out-patient days was very large. These were performed in a room on the ground-floor set apart for the purpose, and called the bleeding-room. The floor was covered with red floorcloth. There was a long movable deal bench, without any back to it, generally placed across the room, facing the windows. On this the patients sat to be bled. On the days when the bleedings were numerous, the pupil for the week would often arrange five or six of these patients in a row, side by side; first fix a bandage round the arm of each, and give them a pewter bleeding-dish to hold in the other hand; then, beginning at one end, open the vein of each in succession, and, when finished with the last, go back to the first, ready to remove the bandage and tie up the arm. It sometimes happened that one of the patients fainted and fell off the bench, and the blood was spilt over the room and the dress of the patient. I do not remember that any other evil often followed this rough mode of venesection. Tooth-drawing was also performed in this room, by the pupil for the week or his substitute, for all applicants. This was often done in a very rough way. The old key was the instrument nearly always used in the extraction of molars, very often applied with more force than skill.

The pupils generally looked upon the bleeding as a drudgery, and complained of the expense of having their lancets ground or set. I have seen one or two of them strop their lancets on the sole of their boots. The pupil for the week was very glad to get a substitute when he could, and in this way I had a large practice in bleeding whilst I was a house-pupil. I think I must often have bled twenty or more in a day. I began bleeding when I had been at the Infirmary not more than six weeks or two months, although I was very timid about it at first. I had also opportunities of performing many of the minor operations of surgery, and even reducing dislocations of the shoulder, elbow, or fingers, without any assistant but the nurse, when I was in the temporary charge of casualties as *locum tenens* for the pupil in charge. In fact, I learned much more of practical surgery and anatomy, and had more dissection, at the Infirmary than at the London School and Hospital I afterwards attended. I should here record one fact connected with getting subjects for dissection. In addition to the opportunities for dissection in the dead-room, in making *post mortem* examinations, and the surreptitious getting of subjects for Dr. Wallis's lectures from the Infirmary, the practice of resurrectionists had been carried on by some of the Infirmary pupils before my time. Two such expeditions were undertaken whilst I was there; but I did not join in either of them. In the first of these one of the house-pupils, who slept in the same room with me (not Mr. Butter), and several others formed a plan to get the body of a pauper, who had been buried in a village churchyard in Somersetshire, five or six miles from Bristol. They hired a horse and gig from a livery stableman, who, I fancy, guessed their purpose and gave information. However, they carried out their plan, took up the body, and brought it to Bristol in a sack in the gig. Unfortunately they broke one of the shafts, and most of them were obliged to walk home. When they came to the Stone Bridge they found a *posse* of watchmen waiting for them, who took them and their cargo into custody, all but my bedroom companion, who was walking behind the rest and escaped. He startled me by coming in through the window of the bedroom in the early morning, covered with dirt, very tired and disgusted with the results of the expedition. Those who were taken were brought before the magistrates during the day, when they were sharply reprimanded, and made to pay the cost of the body being taken back to the village and reburied. But this was the only penalty inflicted on them.

The other resurrectionist attempt I remember was also unsuccessful, and rather ludicrous in its results. Two separate parties of the pupils had formed a plan for taking up the body of a man which had been buried in the Infirmary private cemetery. They met at night at the burying-ground, but could not agree to act together. Some altercation arose, which gave an alarm and put an end to the attempt to get the body; but not to the hostile feelings of the two parties, which had to be appeased by some sort of pugilistic encounter some days after.

I think there were no further attempts at this mode of getting subjects for dissection.

